# CXCL9 secreted by tumor-associated dendritic cells up-regulates PD-L1 expression in bladder cancer cells by activating the CXCR3 signaling

**DOI:** 10.1186/s12865-020-00396-3

**Published:** 2021-01-06

**Authors:** Weigang Xiu, Jingjing Luo

**Affiliations:** 1 Department of Thoracic Oncology and State Key Laboratory of Biotherapy, Cancer Center, West China Hospital, Sichuan University, Chengdu, 610041 PR China; 2 Department of Laboratory Medicine, West China Second University Hospital, Sichuan University, Chengdu, 610041 PR China; 3 Key Laboratory of Birth Defects and Related Diseases of Women and Children (Sichuan University), Ministry of Education, Chengdu, 610041 PR China

**Keywords:** Tumor-associated dendritic cells, Bladder cancer, Programmed death-ligand 1, CXCL9/CXCR3, STAT3/AKT

## Abstract

**Background:**

Tumor-associated dendritic cells (TADCs) can interact with tumor cells to suppress anti-tumor T cell immunity. However, there is no information on whether and how TADCs can modulate programmed death-ligand 1 (PD-L1) expression by cancer cells.

**Methods:**

Human peripheral blood monocytes were induced for DCs and immature DCs were cultured alone, or co-cultured with bladder cancer T24 or control SV-HUC-1 cells, followed by stimulating with LPS for DC activation. The activation status of DCs was characterized by flow cytometry and allogenic T cell proliferation. The levels of chemokines in the supernatants of co-cultured DCs were measured by CBA-based flow cytometry. The impacts of CXCL9 on PD-L1, STAT3 and Akt expression and STAT3 and Akt phosphorylation in T24 cells were determined by flow cytometry and Western blot.

**Results:**

Compared with the control DCs, TADCs exhibited immature phenotype and had significantly lower capacity to stimulate allogenic T cell proliferation, particularly in the presence of recombinant CXCL9. TADCs produced significantly higher levels of CXCL9, which enhanced PD-L1 expression in T24 cells. Pre-treatment with AMG487 abrogated the CXCL9-increased PD-L1 expression in T24 cells. Treatment with CXCL9 significantly enhanced STAT3 and Akt activation in T24 cells.

**Conclusions:**

TADCs produced high levels of CXCL9 that increased PD-L1 expression in bladder cancer T24 cells by activating the CXCR3-related signaling. Our findings may shed new lights in understanding the regulatory roles of TADCs in inhibiting antitumor T cell responses and promoting tumor growth.

## Background

Dendritic cells (DCs) are the most potent antigen-presenting cells to induce an antigen-specific anti-tumor immunity [1]. Activation of DCs can exhibit inflammatory and tolerized phenotypes, dependent the activation conditions. DCs in a tumor environment usually display suppressive and dysfunctional phenotypes, which contribute to the evasion of cancer cells from host immunosurveillance [2]. Tumor-associated DCs (TADCs) can secrete numerous types of cytokines, such as IL-10 and TGF-β1 that inhibit T cell activation and promote tumor cell growth [3]. Actually, IL-10 secreted by tolerized or type 2 DCs suppresses effector T cell function and cytotoxic lymphocyte response [4]. Our previous study has shown that most TADCs exhibit an immature phenotype, such as lower co-stimulatory molecule expression and prone to apoptosis [5]. However, the function of TADCs in the growth of tumors and chemokine expression has not been fully understood.

CXCL9 is one member of the ELR-negative CXC chemokine subfamily, and can be induced by interferon-γ (IFN-γ) [6]. CXCL9 binds to its receptor CXCR3 and can recruit CXCR3+ cells, such as effector T cells, regulatory T cells (Tregs) and CD8+ cytotoxic T cells. Furthermore, TADCs produce high levels of CXCL9, and reduce their antigenicity to induce T cells proliferation [4, 5]. However, it is unclear how CXCL9-induced evasion of cancer cells from host immunosurveillance. In addition, higher frequency of CXCR3+ regulatory T cells in ovarian tumors can inhibit effector T responses to promote tumor progression [7]. In contrast, CXCR3-dependent anti-tumor activity has been found in vitro *and* in vivo and CXCL11 through the CXCR3 can attract CD8+ cytotoxic T cells to inhibit tumor growth [8]. Hence, CXCR3 has a dual role as an oncogenic factor and tumor suppressor [9, 10]. Currently, the precise role of CXCR3 in a specific type of tumor remains in debate [11, 12]. Thus, further investigation of the roles of the CXCL9/CXCL10/CXCL11-CXCR3 signaling is necessary for understanding its regulatory functions, particularly in a tumor environmrnt.

Programmed death ligand-1 (PD-L1) can promote tumor progression by attenuating the function of antigen presenting cells and effector T cells although the precise mechanisms underlying the regulatory role of PD-L1 are still unclear [13]. Previous studies have shown that PD-L1 can inhibit TADC activation and immune function by interfering with DC maturation, leading to difficulty of TADCs to effectively present tumor antigens for inducing T cell immunity [14, 15]. Moreover, PD-L1 over-expression on TADCs was also associated with the reduced expression of immunostimulatory cytokines, co-stimulatory molecules, resulting in T cell anergy [16] and PD-1 paralyzes TADCs by inactivating the NF-κB signaling [17]. Therefore, inhibiting the PD-L1/PD-1 signaling may improve the capability of TADCs to enhance cytotoxic T cell responses [18]. However, there currently is no information on whether TADCs can regulate PD-L1 expression in tumor cells [14].

In the present study, we employed a co-culture experimental system to explore whether TADCs could modulate PD-L1 expression in bladder cancer cells and relevant mechanisms.

## Methods

### Cell culture

The SV-40 immortalized human urothelial cell 1 line (SV-HUC-1) and human bladder cancer T24 cells were obtained from Chinese Academy of Sciences Cell Bank (CASCB, China) and their authenticity was defined by STR. The SV-HUC-1 and T24 cells were cultured in Ham’s F-12 medium (Sigma, USA) and RPMI-1640 medium containing 10% FBS (Gibco, USA), respectively, in a humidified atmosphere containing 37 °C and 5% CO_2_. In some experiments, T24 cells were pre-treated with, or without, 1 μM AMG487 (Tocris, USA) for 2 h and treated with 100 ng/ml of CXCL9 (Peprotech, USA). The control cells were cultured with medium alone.

### Preparation of DCs

Human peripheral blood samples were obtained from healthy individuals and mononuclear cells (PBMCs) were isolated by Ficoll-Hypaque density gradient centrifugation. To generate immature DCs, PBMCs (5.0 × 10^6^ cells/2 ml) were cultured in 6-well plates (Greiner Bio-One, Kremsmünster, Austria) in AIM-V medium containing 10% of FBS (Gibco) for 2 h. After removal of non-adherence cells, the adhered cells were treated with 50 ng/ml of GM-CSF and 50 ng/ml of rhIL-4 (Peprotech) in complete AIM-V medium for 6 days. The cells were exposed to fresh medium with cytokines on day 3 and 5.

### Co-cultured immature DCs with T24 cells

The iDCs (1.0 × 10^6^ cells/well) were co-cultured in triplicate with T24 or SV-HUC-1 cells (1.0 × 10^6^ cells/well) for 24 h and stimulated with 1 μg/ml of LPS (Sigma) for 24 h. Their supernatants were harvested and centrifuged. T24 cells (1.0 × 10^7^ cells /well) were pre-treated with or without, 1 μM AMG487 for 2 h and treated in triplicate with, or without, the supernatants of cultured DCs (no dilution) for 24 h to stimulate PD-L1 expression.

### Purified DCs

After co-cultured with iDCs for 24 h, iDCs were purified using microbeads in a Blood Dendritic Isolation kit II MiltenyiBiotec (Germany), as our previous study [4].

### Mixed lymphocyte response

Human peripheral blood CD3^+^ T cells were purified using Pan T cell Isolation kit (Miltenyi Biotec), as our previous study [5]. The purified DCs (with a purity of > 90%) were irradiated for 30 G by X-irradiator (Gammacell 40 Exactor; MDS Nordion International, USA) for 30 min. The purified CD3^+^ T cells (with purity of > 95%) (2.0 × 10^5^ cells/well) were stimulated in triplicate with the purified iDCs (1.0 × 10^4^ cells/well) in the presence of T24 or recombinant CXCL9-treated T24 cells for 5 days. The T cell proliferation was observed under a light microscope and tested using a CCK-8 kit (Dojindo, Japan).

### Flow cytometry

After co-cultured with iDCs or stimulated, T24 cells were harvested and stained in triplicate with fluorescence-conjugated anti-PD-L1 or isotype control. The intensity of anti-PD-L1 staining was quantified by flow cytometry (CytoFLEX; Beckman Coulter, Brea, USA) and data were analyzed using CytExpert 1.0 software (Beckman Coulter).

Similarly, after co-cultured and LPS stimulation, the mature DCs were isolated and stained with fluorescence-labeled anti-CD86, anti-CD11c, anti-HLA-DR or isotype control (eBioscience). The fluorescent signals were analyzed by flow cytometry.

### TADCs-derived chemokine assays

The levels of IP-10, MCP-1, CXCL9, RANTES and IL-8 in the supernatants of co-cultured DCs were quantified by flow cytometry using Human Chemokine CBA kit (BD Bioscience, USA), according to the manufacturer’s instruction. The data were analyzed by FCAP Array software (BD Bioscience).

### Western blot analysis

T24 cells (2.0 × 10^5^/well) were cultured overnight and stimulated with, or without, CXCL9 for 72 h. The cells were lyzed in lysis buffer and the cell lysates (50 μg/lane) were separated by SDS-PAGE and transferred onto nitrocellulose membranes. The membranes were incubated with anti-Stat3, anti-Atk, anti-pStat3, anti-pAkt, or anti-β-actin (Abcam, UK) overnight and after being washed, the bound antibodies were detected HRP-conjugated second antibodies, followed by visualized using the LI-COR’s Odyssey Infrared Imaging System (LI-COR Biotechnology, USA). The data were quantified by densitometric analysis using ImageJ software.

### Statistical analysis

Data are present as the means ± standard deviation (SD). The difference among groups was analyzed by one-way analysis of variance (ANOVA) and post hoc least significant difference test using SPSS 17.0 software (SPSS, Chicago, USA). A *P*-value of 0.05 was considered statistically significant.

## Results

### Co-culture of immature DCs with bladder cancer T24 cells inhibits DC activation induced by lipopolysaccharide (LPS)

**Fig. 1:**
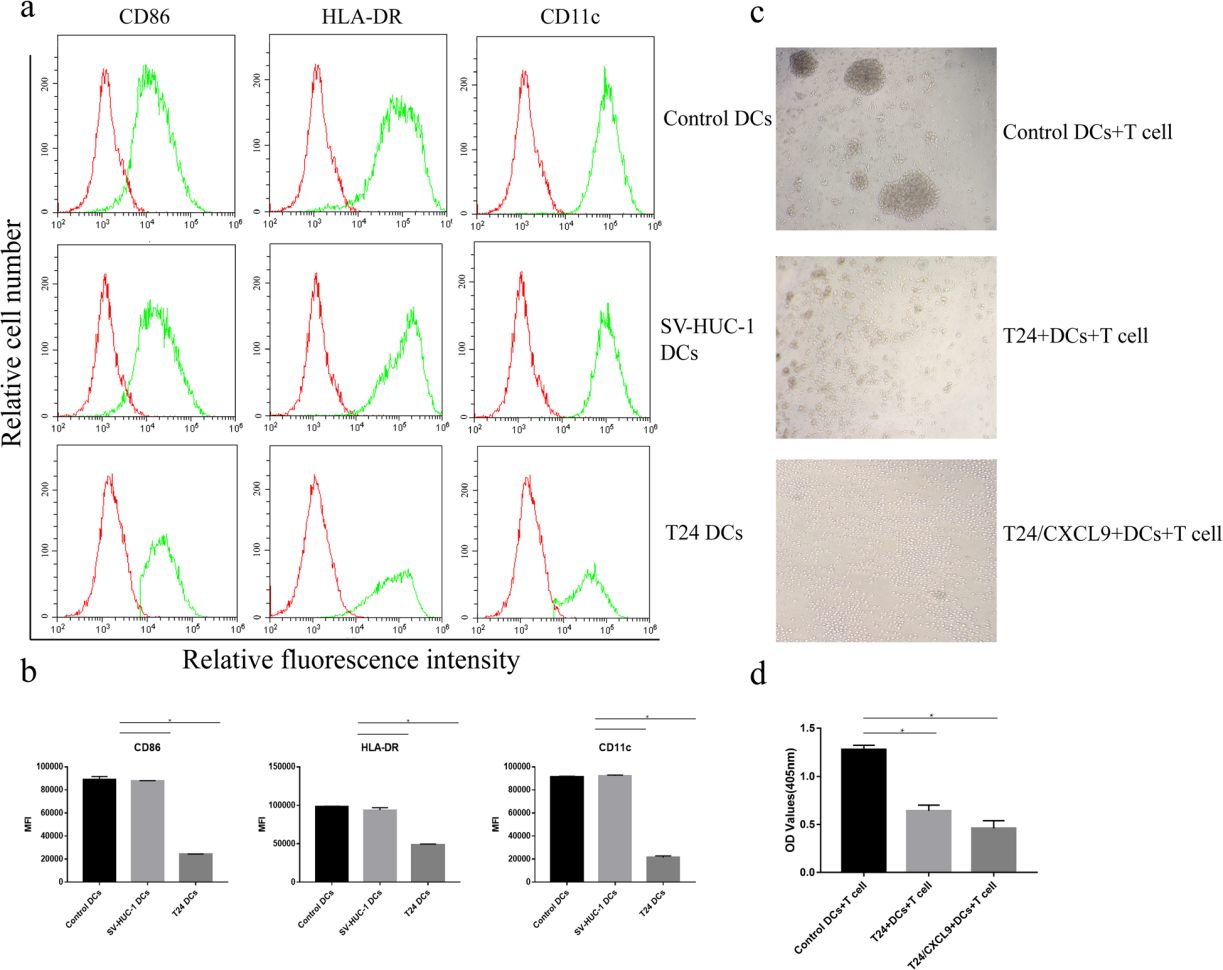
Bladder cancer T24 cells inhibit the LPS-induced DC activation and DC-stimulated T cell proliferation in vitro. Human peripheral blood monocytes were induced for DC differentiation and immature DCs were co-cultured with, or without, T24 or SV-HUC-1 for 24 h, followed by stimulation with LPS for 24 h. The levels of CD86, HLA-DR and CD11c expression in DCs were determined by flow cytometry. The capacity of each group of DCs in the presence of absence of T24 or CXCL9-treated T24 to stimulate the proliferation of purified human CD3 T cells from other subjects was examined by CCK-8 assays. Data are representative flow histograms, images (magnification × 200) or expressed as the mean ± SD of each group from three separate experiments. **a**, **b** Flow cytometry analysis of DC activation. **c**, **d** Microscopy of T cell clones and CCK-8 determined the proliferation of each group of T cells. *P < 0.05

We first induced human monocytes into immature DCs, which were co-cultured with, or without, T24 or SV-HUC-1 for 24 h, followed by stimulating with LPS for DC activation for 24 h. Subsequently, we characterized DC activation by flow cytometry analysis of activation-related CD86, HLA-DR and CD11c expression. As shown in Fig. 1a, DCs after co-culture with SV-HUC-1 displayed high levels of CD86 and HLA-DR as well as CD11c expression, similar to that in the control DCs without co-culture, indicating LPS-induced DC activation. In contrast, TADCs (after co-culture with T24 cells) exhibited lower levels of CD86, HLA-DR and CD11c expression, a hallmark of impaired DC activation (Fig. 1b). Next, we tested the capacity of activated DCs to stimulate allogenic CD3+ T cell proliferation in vitro. We found that control DCs stimulated strong T cell proliferation, which was significantly mitigated by addition of T24 cells and further reduced by addition of CXCL9-treated T24 cells (*p* < 0.05, Fig. 1c and d). Evidently, there was a few of smaller T cell clones after stimulation with T24 + DCs and much less after stimulation with CXCL9-treated T24 + DC, relative to that of control DCs (Fig. 1c and d). These two lines of data indicated that co-culture with bladder cancer T24 cells inhibited the LPS-induced immature DC activation and their capacity to prime allogenic T cell proliferation in vitro.

### Bladder cancer T24 cells promote the production of CXCL9 by DCs

**Fig. 2:**
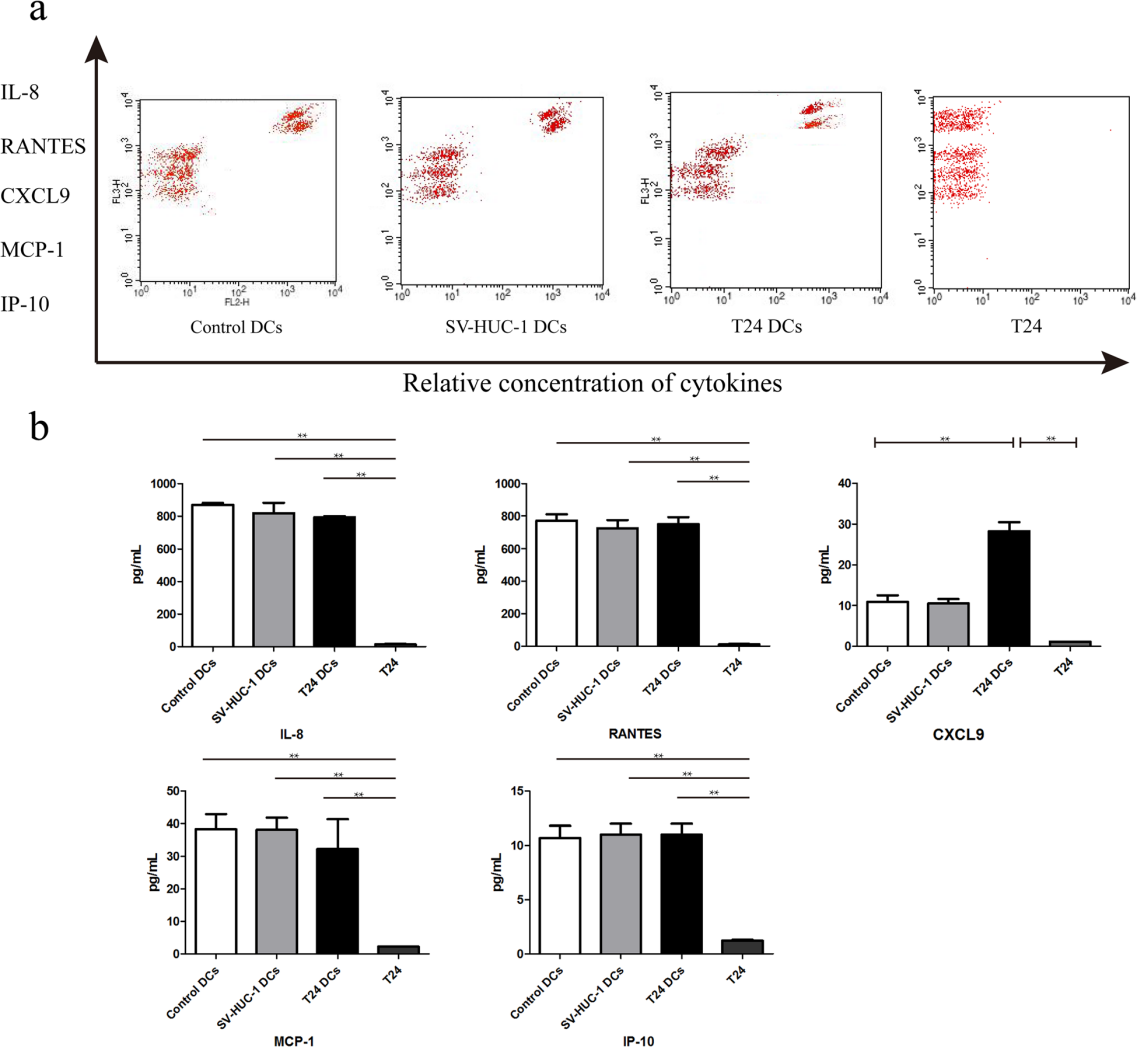
Co-culture with T24 cells enhances the production of CXCL9 in DCs. Following co-culture of immature DCs with, or without, T24 or SV-HUC-1 cells as well as T24 cells alone for 24 h, the levels of IL-8, RANTES, CXCL9, MCP-1 and IP-10 in the supernatants of cultured cells were quantified by flow cytometry using a Cytometric Bead Array (CBA) kit. Data are representative flow histograms or expressed as the mean ± SD of each group of cells from three separate experiments. **a** Flow cytometry analysis. **b** Quantitative analysis. *P < 0.05, **P < 0.01

Chemokines are crucial for promoting anti-tumor immunity [19]. To understand how T24 cells affected the LPS-induced DC activation, we measured the levels of IL-8, RANTES, CXCL9, MCP-1and IP-10 in the supernatants of co-cultured cells by Cytometric Bead Array-based flow cytometry. There was no significant difference in the levels of any chemokine tested between the control DCs and DCs co-cultured with SV-HUC-1 and there were very low levels of cytokines tested in the supernatants of cultured T24 cells (Fig. 2). The levels of CXCL9, but not IL-8, RANTES, MCP-1 and IP-10, in the supernatants of TADCs were significantly higher than that in the control DCs (Fig. 2). Hence, bladder cancer T24 cells enhanced the production of CXCL9 by the LPS-treated DCs.

### CXCL9 up-regulates PD-L1 expression in bladder cancer T24 cells through its receptor CXCR3

**Fig. 3:**
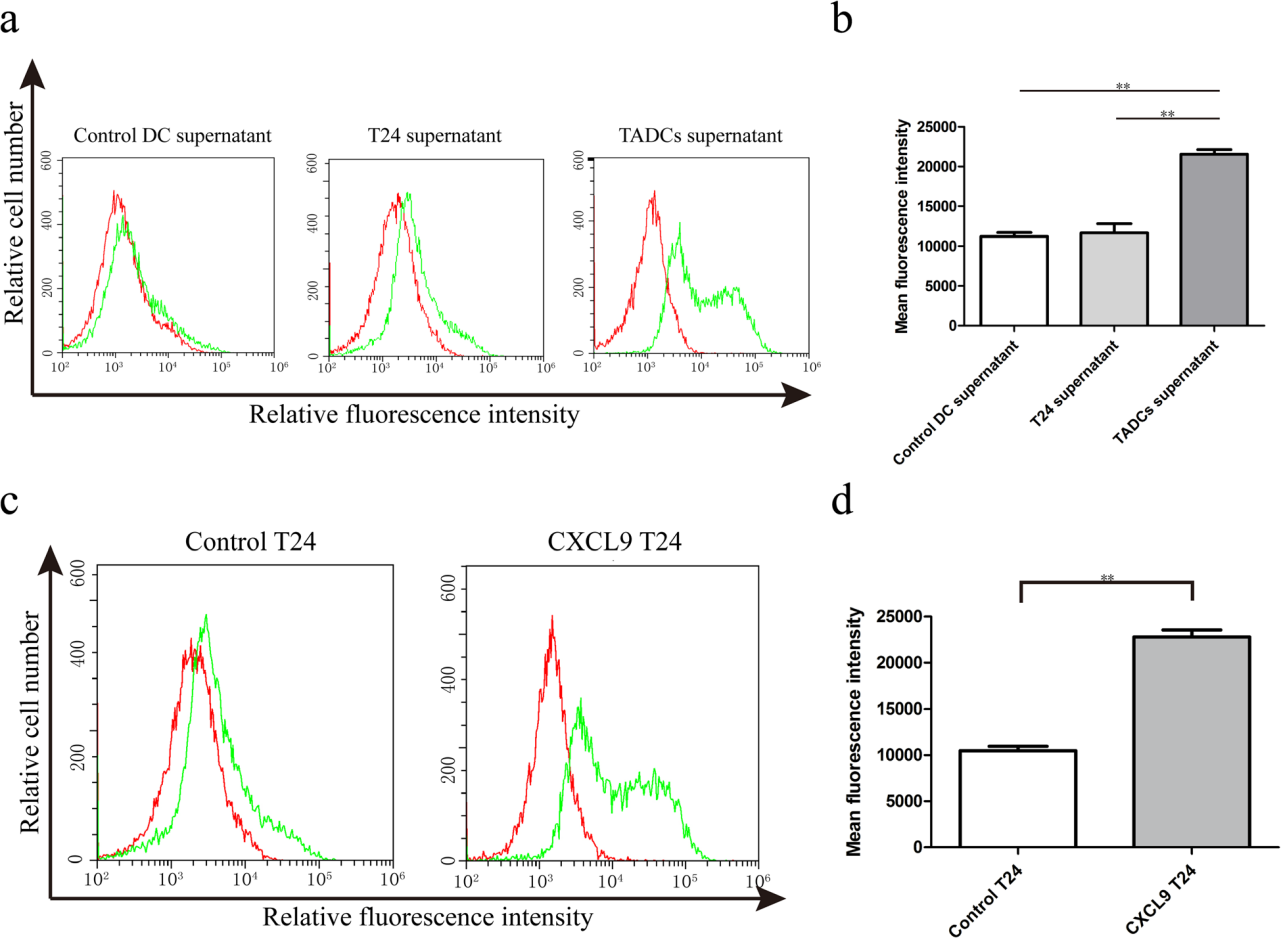
CXCL9 up-regulates PD-L1 expression in T24 cells. T24 cells were treated with the supernatants from cultured DCs alone, T24 alone or co-cultured TADCs in the presence or absence of additional recombinant CXCL9 (100 ng/mL). The levels of PD-L1 in individual groups of T24 cells were quantified by flow cytometry using specific anti-PD-L1. Data are representative flow histograms or expressed as the mean ± SD of each group of cells from three separate experiments. **a**, **c** Flow cytometry analysis. **b**, **d** Quantitative analysis. **P < 0.01

**Fig. 4:**
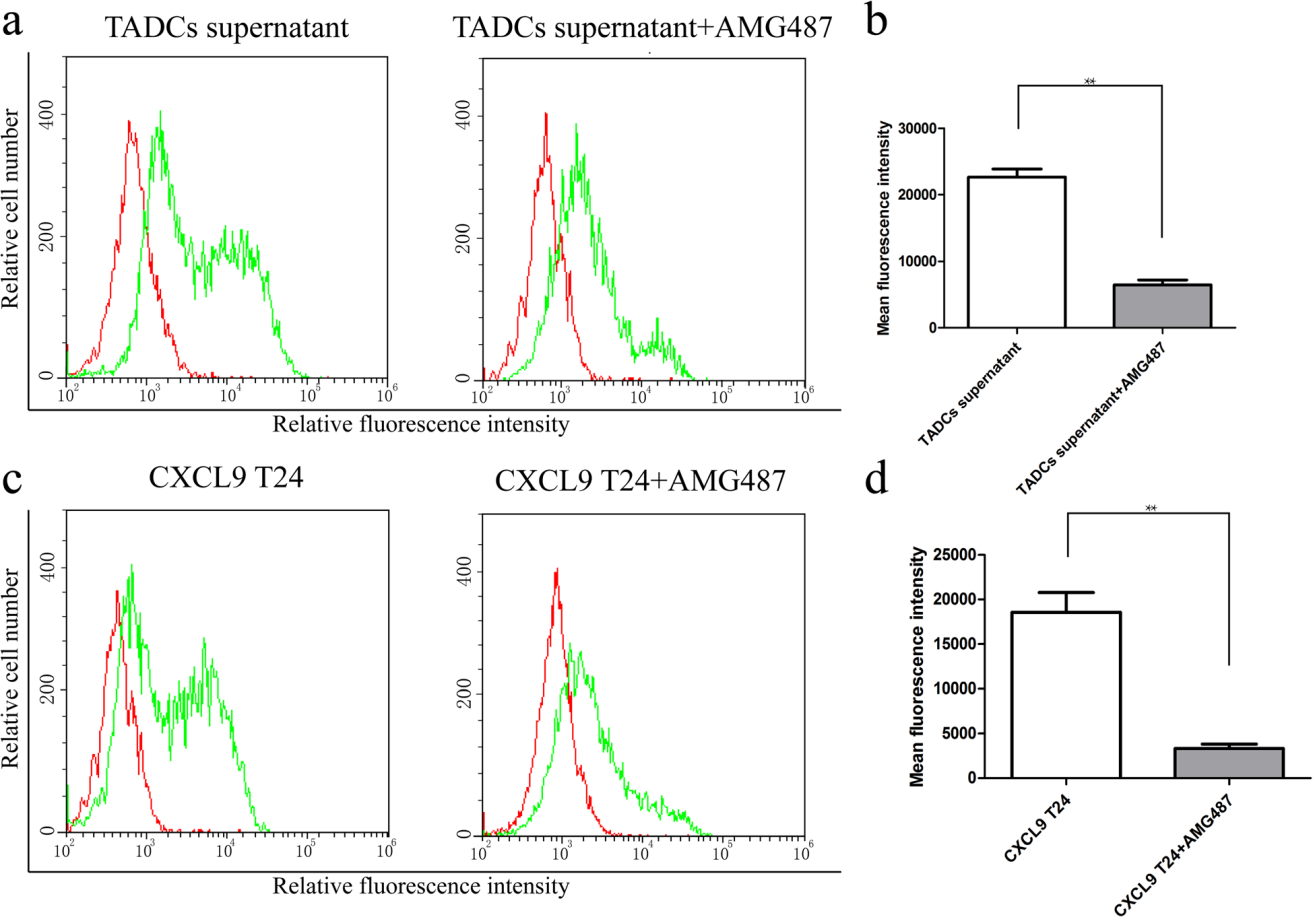
Pre-treatment with CXCR3-specific antagonist AMG487 abrogates the CXCL9-up-regulated PD-L1 expression in T24 cells. T24 cells were pre-treated with, or without, AMG487 (1 μM) for 2 h and treated with the supernatants of co-cultured TADCs or CXCL9 (100 ng/mL) for 72 h**.** The levels of PD-L1 in individual groups of T24 cells were quantified by flow cytometry using specific anti-PD-L1. Data are representative flow histograms or expressed as the mean ± SD of each group of cells from three separate experiments. **a**, **c** Flow cytometry analysis. **b**, **d** Quantitative analysis. *P < 0.05, **P < 0.01

PD-L1 is an important suppressor of anti-tumor T cell immunity [20]. To explore the consequence of up-regulated CXCL9 on T24 cells, we tested whether the up-regulated CXCL9 could modulate PD-L1 expression in T24 cells. We found that treatment with the supernatants of TADCs significantly increased the levels of PD-L1 expression in T24 cells, relative to that in the T24 cells treated with the supernatants from the cultured T24 or control DC (Fig. 3a and b). Similarly, treatment with recombinant CXCL9 also significantly increased the levels of PD-L1 expression in T24 cells (Fig. 3c and d). More importantly, pre-treatment with the CXCR3 antagonist AM487 abrogated the supernatant and CXCL9-enhanced PD-L1 expression in T24 cells (Fig. 4). These data clearly indicated that CXCL9 in the supernatants of co-cultured TADCs promoted PD-L1 expression in T24 cells through the CXCR3 receptor.

### CXCL9 enhances the STAT3 and AKT activation in bladder cancer T24 cells

**Fig. 5:**
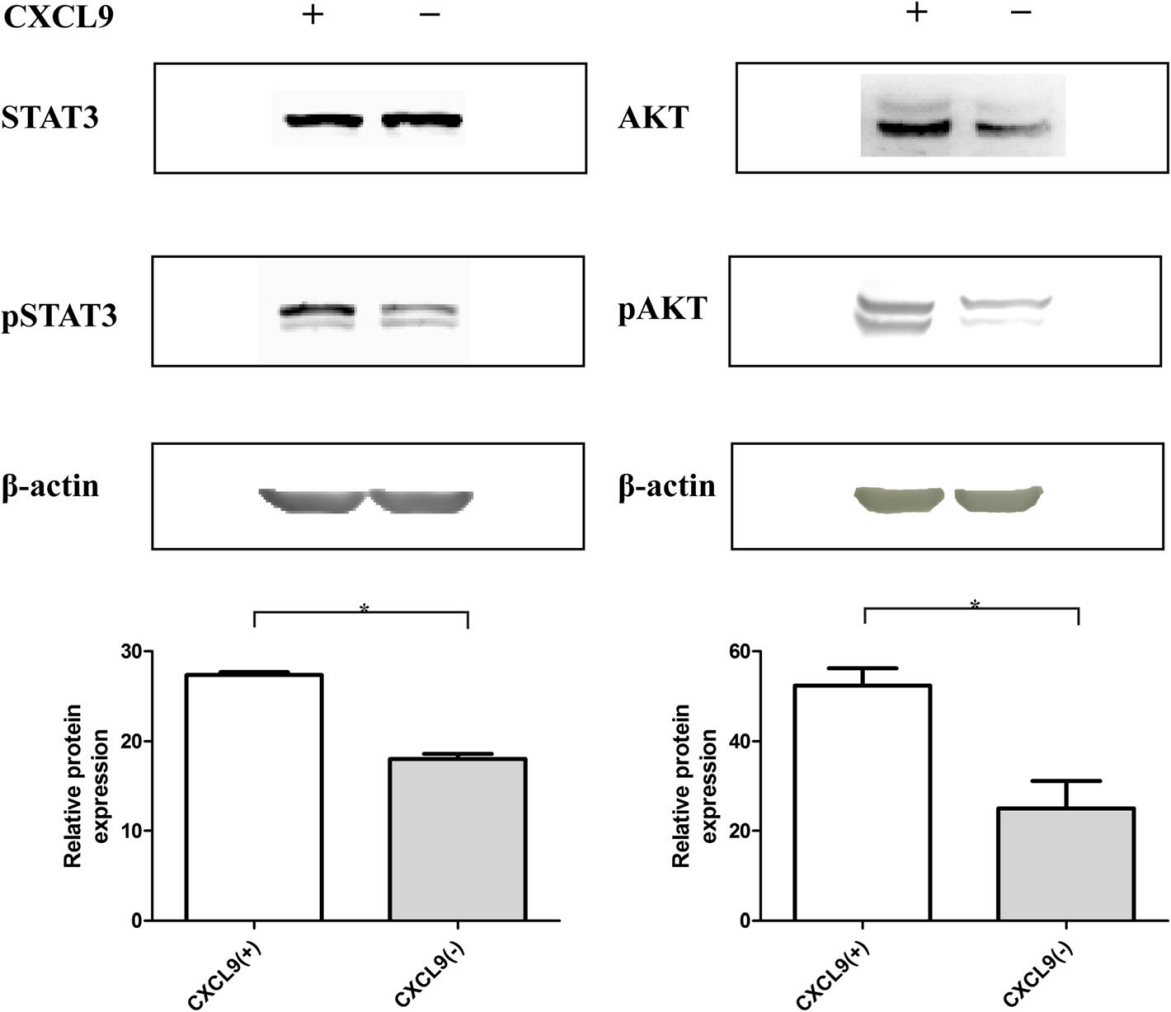
CXCL9 treatment enhances the STAT3 and Akt activation in T24 cells. T24 cells were treated with, or without, CXCL9 for 72 h and the relative levels of STAT3 and Akt expression and phosphorylation were quantified by Western blot. Data are representative images or expressed as the mean ± SD of each group of cells from three separate experiments. **a** Western blot analysis. **b** Quantitative analysis. *P < 0.05

CXCL9 binds to the CXCR3, leading to increased intracellular calcium levels that activate multiple signal pathways, such as the Jak/Stat and PI3K/Akt signaling, during the development of tumors [21]. To further understand how the increased CXCL9 expression by TADCs regulated PD-L1 expression in bladder cancer T24 cells, we tested the effect of CXCL9 treatment on the expression of STAT3 and Akt activation in T24 cells by Western blot. We found that treatment with recombinant CXCL9 significantly increased the relative levels of STAT3 and Akt expression and phosphorylation in T24 cells (Fig. 5). The enhanced STAT3 and Akt signaling by CXCL9 may contribute to its role in up-regulating PD-L1 expression in T24 cells.

## Discussion

DCs are professional antigen presenting cells and can effectively present antigen determinants to activate T cells because they express high levels of co-stimulators and MHC molecules, such as CD80, CD86 and MHC II, particularly after activation [22, 23]. In this study, we found that T24-exposed DCs displayed significantly reduced levels of CD86, HLA-DR, CD11c expression, and exhibited an impaired capacity to stimulate allogenic T cell proliferation, particularly for those exposed to the CXCL9-treated T24 cells. These data indicated that T24 inhibited the LPS-induced DC activation, consistent with previous studies [24, 25] and support the notion that tumor cells can suppress the differentiation, trafficking and activation of DCs in a tumor microenvironment for their evasion from immunosurveillance [26]. There is growing evidence that TADCs change their function from immunostimulatory to immunosuppressive types during the tumor progression [27], which may be attributed to high levels of PD-L1/PD-1 expression in the tumor microenvironment [3, 17, 18]. Interestingly, we found that TADCs (after co-cultured with T24 cells) produced significantly higher levels of CXCL9, but not IL-8, RANTES, MCP-1 and IP-10. Furthermore, treatment with the supernatants of cultured TADCs, or recombinant CXCL9 significantly up-regulated PD-L1 expression in T24 cells, which were abrogated by pre-treatment with CXCR3-specific antagonist AM487. In addition, treatment with CXCL9 significantly enhanced the activation of STAT3 and Akt in T24 cells. Such novel findings indicated that bladder cancer cells enhanced CXCL9 in TADCs, which significantly up-regulated PD-L1 expression in cancer cells by activating the CXCR3-related STAT3/Akt signaling. Because the PD-L1 is a potent inhibitor of antitumor T cell immunity the up-regulated PD-L1 expression in cancer cells should inhibit the activation and function of antigen presenting cells and effector T cells, promoting cancer evasion from antitumor responses. Given that anti-PD-1 and anti-PD-L1 are promising to treat some types of solid cancers the CXCL9/CXCR3/PD-L1 axis may be new therapeutic targets for intervention of bladder cancers. Therefore, our findings may provide new insights into the mechanisms underlying how tumors escape from immunosurveillance.

Many types of solid cancers have a complex chemokine network, which can hijack the chemokine system from immune cells and promote their own growth and metastasis in autocrine and paracrine manners [28, 29]. For instance, some chemokines from tumor cells can alter DC’s phenotype and impair immune response [26, 29, 30]. CXCR3 plays a crucial role in tumor microenvironment [31] and CXCR3 over-expression can recruit more TADCs [32]. Furthermore, CXCR3 can regulate the growth and metastasis of cancers [33, 34]. High levels of CXCL9 and CXCL10 expression in lymph nodes can promote melanoma cell metastasis through the CXCR3 signaling [35]. Up-regulated CXCR3 expression in primary and metastatic ovarian tumors was associated with a reduced progression-free survival and overall survival [36]. It is well known that IFN-γ can induce the expression of ELR-negative CXC chemokines, such as CXCL9, and enhance PD-L1 expression [37–40]. In this study, we found that CXCL9 secreted by TADCs enhanced PD-L1 expression in bladder cancer T24 cells, which was abrogated by the CXCR3 antagonist AMG487. In addition, we found that CXCL9 treatment up-regulated STAT3 and Akt activation in T24 cells. Such findings suggest that in a tumor environment, IFN-γ can through its IFNR1/2 receptors activate the JAK/STAT3 signaling to directly induce PD-L1 expression and enhance CXCL9 expression, which indirectly through its CXCR3 activates the STAT3 and Akt signaling to up-regulate PD-L1 expression in tumor cells [21, 38, 41]. Actually, phosphorylated STAT1 and STAT3 dimer in tumor cytosol can bind to the PD-L1 promoter to induce PD-L1 expression [42] while inhibition of STAT3 activity by STATTIC mitigates the CXCR3 activation-induced PD-L1 expression in tumor cells [21]. Hence, STAT3 activation is crucial for inducing PD-L1 expression in tumor cells and inhibition of STAT3 activity may inhibit the CXCL9/CXCR3 signaling-induced PD-L1 expression, benefiting bladder cancer patients. Currently, the STAT3 inhibitors are being tested in clinical trials for bladder cancer [43]. Given that up-regulated PD-L1 expression should attenuate antitumor T cell immunity, our findings may shed new lights in the feedback regulation of inflammatory IFN-γ responses on antitumor T cell immunity and tumor evasion from immunosurveillance.

## Conclusions

In summary, our data indicated that co-culture of immature DCs with bladder cancer T24 cells generated TADCs that displayed immature and suppressive phenotypes with less capacity to induce allogenic T cell proliferation. TADCs produced higher levels of CXCL9 that up-regulated PD-L1 expression in T24 cells, dependent on its CXCR3 signaling. CXCL9 treatment enhanced STAT3 and Akt activation in T24 cells. Hence, CXCL9 induced PD-L1 expression, perhaps by activating the CXCR3/STAT3 and Akt signaling. Therefore, our findings suggest that the CXCL9/CXCR3/PD-L1 axis may be the potential therapeutic targets for bladder cancer.

## Data Availability

The datasets used and/or analyzed during the current study are available from the corresponding author on reasonable request.

## Abbreviations

TADCs: Tuberculosis
iDCs: immature DCs
IFN-γ: INTERFERON-γ
PD-L1: programmed death ligand-1
FBS: Fetal bovine serum
FACS: Fluorescence activated cell sorter
MFI: Mean fluorescence intensity
CBA: Cytometric bead array
SV-HUC-1: SV-40 immortalized human uroepithelial cell 1
PBMCs: Peripheral blood mononuclear cells
