# Correction to: CXCL9 secreted by tumor-associated dendritic cells up-regulates PD-L1 expression in bladder cancer cells by activating the CXCR3 signaling

**DOI:** 10.1186/s12865-021-00408-w

**Published:** 2021-02-25

**Authors:** Weigang Xiu, Jingjing Luo

**Affiliations:** 1 Department of Laboratory Medicine, West China Second University Hospital, Sichuan University, Chengdu, 610041 PR China; 2 Key Laboratory of Birth Defects and Related Diseases of Women and Children (Sichuan University), Ministry of Education, Chengdu, 610041 PR China; 3 Department of Thoracic Oncology and State Key Laboratory of Biotherapy, Cancer Center, West China Hospital, Sichuan University, Chengdu, 610041 PR China

**Correction to: BMC Immunol (2021) 22:3**

**https://doi.org/10.1186/s12865-020-00396-3**

It was highlighted that the original article [1] contained errors in the authors affiliations. This Correction article shows the incorrect and correct author affiliations. The original article has been updated.

**Incorrect**

Weigang Xiu^1^ and Jingjing Luo^2,3^

1 Department of Thoracic Oncology and State Key Laboratory of Biotherapy, Cancer Center, West China Hospital, Sichuan University, Chengdu 610041, PR China. 2 Department of Laboratory Medicine, West China Second University Hospital, Sichuan University, Chengdu 610041, PR China. 3 Key Laboratory of Birth Defects and Related Diseases of Women and Children (Sichuan University), Ministry of Education, Chengdu 610041, PR China.

**Correct**

Weigang Xiu^1,2,3^ and Jingjing Luo^1,2^

1 Department of Laboratory Medicine, West China Second University Hospital, Sichuan University. 2 Key Laboratory of Birth Defects and Related Diseases of Women and Children (Sichuan University), Ministry of Education. 3 Department of Thoracic Oncology and State Key Laboratory of Biotherapy, Cancer Center, West China Hospital, Sichuan University, Chengdu 610041, PR China.
